# Technology and its clinical application in the field of computer-assisted radiology and surgery

**DOI:** 10.2349/biij.3.3.e41

**Published:** 2007-07-01

**Authors:** K Inamura, HU Lemke

**Affiliations:** 1 Kansai University of International Studies, Miki-city, Hyogo, Japan; 2 Technical University Berlin, Electrical Engineering and Computer Science, Berlin, Germany

**Keywords:** Computer-assisted radiology and surgery, CARS, TIMMS

## Abstract

The field of computer-assisted radiology and surgery involves a wide spectrum of topics based on medicine, physics, computer science and even sociology. The progress of development and recent trends in this field is described in this paper. Firstly, the chronological change in presented papers in past international conferences of Computer Assisted Radiology and Surgery (CARS) from 1985 to 2007 is illustrated in terms of topics, which are grouped into six main categories. Secondly, new directions and related topics are described by means of an example of a digital operating room. Problems in the operation room (OR) and solution concepts are pointed out while a therapy imaging and model management system (TIMMS) is presented as a possible solution. Finally, patient modelling related topics for CARS are listed.

## TOPICS AND THEIR CHRONOLOGICAL CHANGES

The International Congress and Exhibition of Computer Assisted Radiology and Surgery (CARS) involves a wide spectrum of topics based on medicine, physics, computer science and even sociology.

From the first CARS in 1985 [1, 2] until the 21st CARS in 2007, more than 60 topics [3, 4] have been presented at the congress. In general, these topics provided the basis for the titles of the sessions. With respect to clinical applications these topics may be grouped into six main categories, i.e. A to F, as below.

### A. Surgery, orthopedics, maxillofacial and other invasive-related topics

1. Surgery, anthropometry, surgical simulator, surgical navigation, neurosurgery, spinal surgery, thoracic abdominal surgery, standards in information-guided therapy, surgical robotics, optical diagnosis and in-situ tissue analysis, ergonomics and motion analysis, curved and steerable instruments and endoluminal.
2. Orthopedics
3. Maxillofacial and implantology
4. Virtual endoscopy
5. Cardio-vascular, coronary and medical innovation and technology

### B. Image processing and display

1. Image processing
2. Display
3. Man-computer interaction and virtual reality
4. Workstation
5. Computer vision and computer graphics

### C. Medical imaging, modalities and their related topics

1. MRI
2. CT
3. Nuclear medicine
4. Ultrasound
5. Digital radiography involving a flat panel detector
6. Angiography involving DSA
7. Mathematical modeling for reconstruction etc
8. Multi-modality imaging

### D. Computer-Aided Diagnosis

1. 1Breast
2. Thoracic
3. Abdomen
4. Brain

### E. Infrastructure

1. PACS, RIS, HIS, electronic medical record, workflow, standards such as DICOM, Integrating Healthcare Enterprise (IHE)
2. Telemedicine
3. Voice recognition application
4. Healthcare infrastructure, interface between medicine and computer sciences, and security
5. Technology assessment and/or social implications
6. Education, knowledge-based systems, expert systems, learning systems and training systems
7. Planning in the radiology department
8. Strategic thinking, decision making and biointelligence

### F. Radiation therapy and minimal invasive therapy

1. Therapeutic workstations
2. Computer-assisted radiotherapy
3. Minimum invasive spinal therapy
4. Image-guided diagnosis and therapy of the prostate

**Figure 1:**
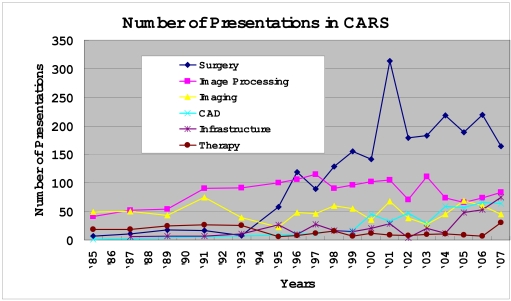
Chronological change of number of presentations in CARS from 1985 to 2007.

The chronological change in the number of presentations with respect to the six categories is shown in Figure 1.

The International Journal of Computer Assisted Radiology and Surgery was founded in 2005, and has been regularly published six times a year [5]. Every year a supplement to the journal is published as the Proceedings of the International Congress and Exhibition of CARS[6].

## NEW DIRECTIONS AND TOPICS OF CARS

Appropriate use of information and communication technology (ICT) and mechatronic (MT) systems is considered by many experts as a significant contribution to improve workflow and quality of care in the Operating Room (OR). Different imaging modalities and a wide spectrum of other information sources need to be digitally integrated to build a suitable patient model. This will require an IT architecture-supporting surgical assist systems, which can be adapted to specific surgical interventions and patient care situations.

## Problems in the OR and solution concepts

Since the OR and image-based interventional suites are the most cost-intensive sector in the hospital, the optimisation of workflow processes has become of particular concern among healthcare providers, managers, and administrators. The understanding and management of workflows should become an integral part in the planning and implementation of complex digital infrastructures that support diagnostic and interventional procedures (i.e. interventional radiology, minimal interventional surgery, computer-assisted surgical procedures and information-guided therapy (IGT)).

This leads to the concept of an ICT-supported OR, which may be named “Therapy Imaging and Model Management System” (TIMMS). A TIMMS, for example as part of a surgical cockpit, should support the essential functions that enable and advance image, and in particular, patient model guided therapy. Within this concept, the traditional image centric world view of the image-guided computer aided diagnosis (CAD) and surgical assist system (SAS) technology is complemented by an IT model-centric world view. Such a view is founded in the special modelling needs of a number of modern surgical interventions as compared to the imaging intensive working mode of diagnostic radiology, from which many surgical assist systems are conceptualised and developed.

**Figure 2:**
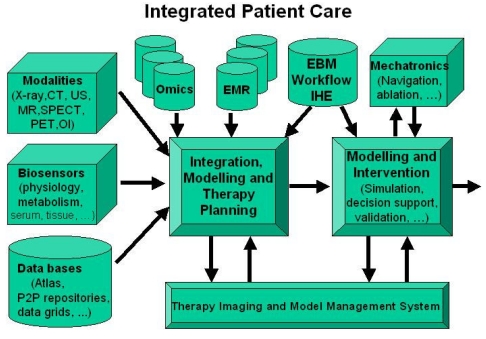
Example of data sources for integrated patient care.

A TIMMS provides the ICT-based infrastructure and component architecture necessary for surgical/interventional workflow management of the modern digital OR. The concept and design of a TIMMS is based on the assumption that significant improvement in the quality of patient care, as well as ergonomic and health-economic progress in the OR can only be achieved by means of an ICT-supported management of intervention-related data, images, information, models and tools. Figure 2 gives an example of how these data sources may be integrated.

New patient modelling methods and tools are required to position TIMSS as the key system for an integrated patient care philosophy. The IT model-centric world view, however, need not only be applied to the patient data, but also to a TIMMS specifically or to any SAS generally.

Adaptability to variable patient situations and differing surgeons’ requirements can only be achieved with systems modelling methods, which take account of processes, states, interfaces and a hierarchy of logical and physical structures. Informatics with its tool set for modelling of discrete digital systems will become an essential discipline for model-guided therapy with adaptive assist systems.

## Therapy imaging and model management system (TIMMS)

The construction of the patient-specific medical model (PSM) will be used as the central construct within a TIMMS, which may be described as an adaptive surgical assist system. Ideally, the PSM, engines and repositories should be integrated into a suitable TIMMS infrastructure to support the planning, execution and validation of an intervention.

Considering software engineering principles, such a system needs to be designed to provide a highly modular structure. Modules may be defined on different granulation levels. The first list of components (e.g. high and low level modules) comprising engines and repositories of an SAS, which should be integrated by a TIMMS, is currently being compiled in a number of R&D institutions.

**Figure 3:**
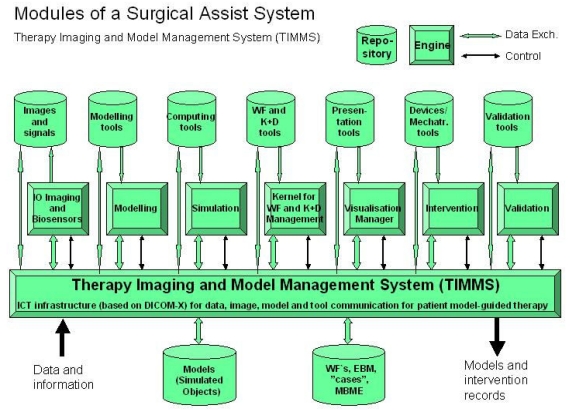
A possible logical structural model of a Therapy Imaging and Model Management System (TIMMS).

Figure 3 shows a concept of a logical structural model (meta architecture) of a high level generic modular architecture in a surgical assist system. The high level modules are abstracted from many specific CAS/IGT systems, which have been developed in recent years. In general, a combination of these can be found in most R&D as well as commercial SAS systems. A central position in Figure 3 is occupied by the “Kernel for workflow and knowledge and decision management”. It provides the strategic intelligence for preoperative planning and intraoperative execution. Often this module (or parts thereof) is integrated into some of the other engines, as the need may have demanded. In any case, adaptation of the therapeutic workflow to an actual patient care situation should be based on the PSM and realized by the Kernel.

## Modelling related topics for CARS

By default, the broader the spectrum of different types of interventional/surgical workflows, which have to be considered, the more effort has to be given for designing appropriate PSM’s and associated services. The following list contains some examples of modelling tools and aspects, derived from different types of surgical workflows, which may have to be considered:

- Geometric modelling including volume and surface representations
- Properties of cells and tissue
- Segmentation and reconstruction
- Biomechanics and damage
- Tissue growth
- Tissue shift
- Prosthesis modelling
- Fabrication model for custom prosthesis
- Properties of biomaterials
- Pharmacokinetics and Pharmacodynamics of normal and pathologic tissue
- Atlas-based anatomic modelling
- Template modelling
- FEM of medical devices and anatomic tissue
- Collision response strategies for constraint deformable objects
- Variety of virtual human models
- Life-like physiology and anatomy
- Modelling of the biologic continuum
- Animated models
- Multi-scale modelling
- Fusion/integration of data/images
- Coordinate systems between different models incl. patient, equipment and OR
- Modelling of workflows

Real-time aspects typically identified for imaging during intervention are equally applicable for the generation and management of these models. Further development of modelling tools, which have been built for CAD purposes, may provide a good basis to facilitate an improved integration of diagnostic and therapeutic applications.

In addition, the development of standards to support the implementations of these new interventional tools must be carried out in parallel. The DICOM Working Group 24 “DICOM in Surgery” has recently been founded to support the implementation of ICT systems in the digital OR [7]. Promotion of research and development projects relating to the above methods, tools and systems is therefore a major challenge for future CARS congresses.

## DISCUSSION

The features of CARS could be stated as follows.

**Interdisciplinary:** CARS bridges the gap between engineering and medicine, with emphasis on the clinical applications of information science and health care technology. It focuses on the interdisciplinary topics included in the content of the categories shown in A to F above.

**Innovation and Flexibility:** CARS is innovation driven and flexible to include hot topics, new ideas, new concepts and different point of views. Sessions have been organised according to problem-oriented thinking. Session names have been revised rather frequently each year compared with other international conferences, because participants of CARS want to adapt appropriate solutions to chronologically changing problems. For example, the topics of “Computer vision” and “Computer graphics”, which emerged in 1985, were revised to “Workstation” from 1991 and to “Image processing and display” from 1995.

**Internationality and Cooperation:** The significance and importance of joint conferences should be pointed out. It was 15 years ago, during his stay at Osaka University as a visiting professor in 1992, that Heinz U Lemke encouraged Kiyonari Inamura to consider the extension of CAR topics, beyond conventional radiology. Lemke’s idea was realized in 1995 when CAR collaborated with EuroPACS, ISPRAD, the International Society for Computer Aided Surgery-ISCAS and Computed Maxillofacial Imaging-CMI.

In 1996, CAR, in collaboration with the International Society for Computer Aided Surgery, was held in Paris. However, the first CAR outside Europe was in Japan in 1998, and that was the beginning of a new era for CARS, which since then collaborated regularly with more than three international formal annual conferences.

Presently, for example in 2007, six international conferences have joined with CARS and benefit from common intellectual property. Efficacy and efficiency of knowledge communication as well as inclusion of a new concept and/or new ideas are the obvious advantages of joint international conferences.

Young scientists who wish to accumulate and digest new knowledge, will especially be stimulated during 4 days in the last week each June, when CARS takes place.

We are confident that in the future, CARS will increasingly contribute to the education and training of young scientists, even though the development of new technologies and highly advanced medical applications are the primary aims of CARS. CARS with its disciplines and interdisciplines will provide a training environment for young scientists to acquire and put into practice new knowledge in computer assisted radiology and surgery.
